# Inflammation versus regulation: how interferon-gamma contributes to type 1 diabetes pathogenesis

**DOI:** 10.3389/fcell.2023.1205590

**Published:** 2023-05-24

**Authors:** David J. De George, Tingting Ge, Balasubramaniam Krishnamurthy, Thomas W. H. Kay, Helen E. Thomas

**Affiliations:** ^1^ Immunology and Diabetes Unit, St Vincent’s Institute, Fitzroy, VIC, Australia; ^2^ Department of Medicine, St Vincent’s Hospital, University of Melbourne, Fitzroy, VIC, Australia

**Keywords:** type 1 daibetes mellitus, interferon gamma (IFNγ), CD8 T cells, inflammation, autoimmunity, T cell proliferation

## Abstract

Type 1 diabetes is an autoimmune disease with onset from early childhood. The insulin-producing pancreatic beta cells are destroyed by CD8^+^ cytotoxic T cells. The disease is challenging to study mechanistically in humans because it is not possible to biopsy the pancreatic islets and the disease is most active prior to the time of clinical diagnosis. The NOD mouse model, with many similarities to, but also some significant differences from human diabetes, provides an opportunity, in a single in-bred genotype, to explore pathogenic mechanisms in molecular detail. The pleiotropic cytokine IFN-γ is believed to contribute to pathogenesis of type 1 diabetes. Evidence of IFN-γ signaling in the islets, including activation of the JAK-STAT pathway and upregulation of MHC class I, are hallmarks of the disease. IFN-γ has a proinflammatory role that is important for homing of autoreactive T cells into islets and direct recognition of beta cells by CD8^+^ T cells. We recently showed that IFN-γ also controls proliferation of autoreactive T cells. Therefore, inhibition of IFN-γ does not prevent type 1 diabetes and is unlikely to be a good therapeutic target. In this manuscript we review the contrasting roles of IFN-γ in driving inflammation and regulating the number of antigen specific CD8^+^ T cells in type 1 diabetes. We also discuss the potential to use JAK inhibitors as therapy for type 1 diabetes, to inhibit both cytokine-mediated inflammation and proliferation of T cells.

## 1 Introduction

Type 1 diabetes (T1D) is an autoimmune disease with onset from early childhood. It is a challenging disease to study mechanistically in humans because the pathology unfolds in the microscopic pancreatic islets that are not usually amenable to study during the time when the disease is most active prior to or at the time of clinical symptomatic onset. The non-obese diabetic (NOD) mouse model, with many similarities to but also some significant differences from human autoimmune diabetes, provides an opportunity, in a single in-bred genotype, to explore pathogenic mechanisms in molecular detail (Thomas et al., 2016).

In T1D, the insulin producing pancreatic *ß* cells are destroyed by autoreactive CD8^+^ T cells. Direct recognition of *ß* cells by CD8^+^ cytotoxic T cells depends on the formation of an immune synapse between the autoreactive TCR on CD8^+^ T cells and a *ß* cell peptide-loaded MHC class I complex. Thus, targeting CD8^+^ T cell mediated destruction of *ß* cells directly, either through regulation of this interaction or by inhibiting the development or cytotoxic function of autoreactive CD8^+^ T cells, may prevent T1D.

**FIGURE 1:**
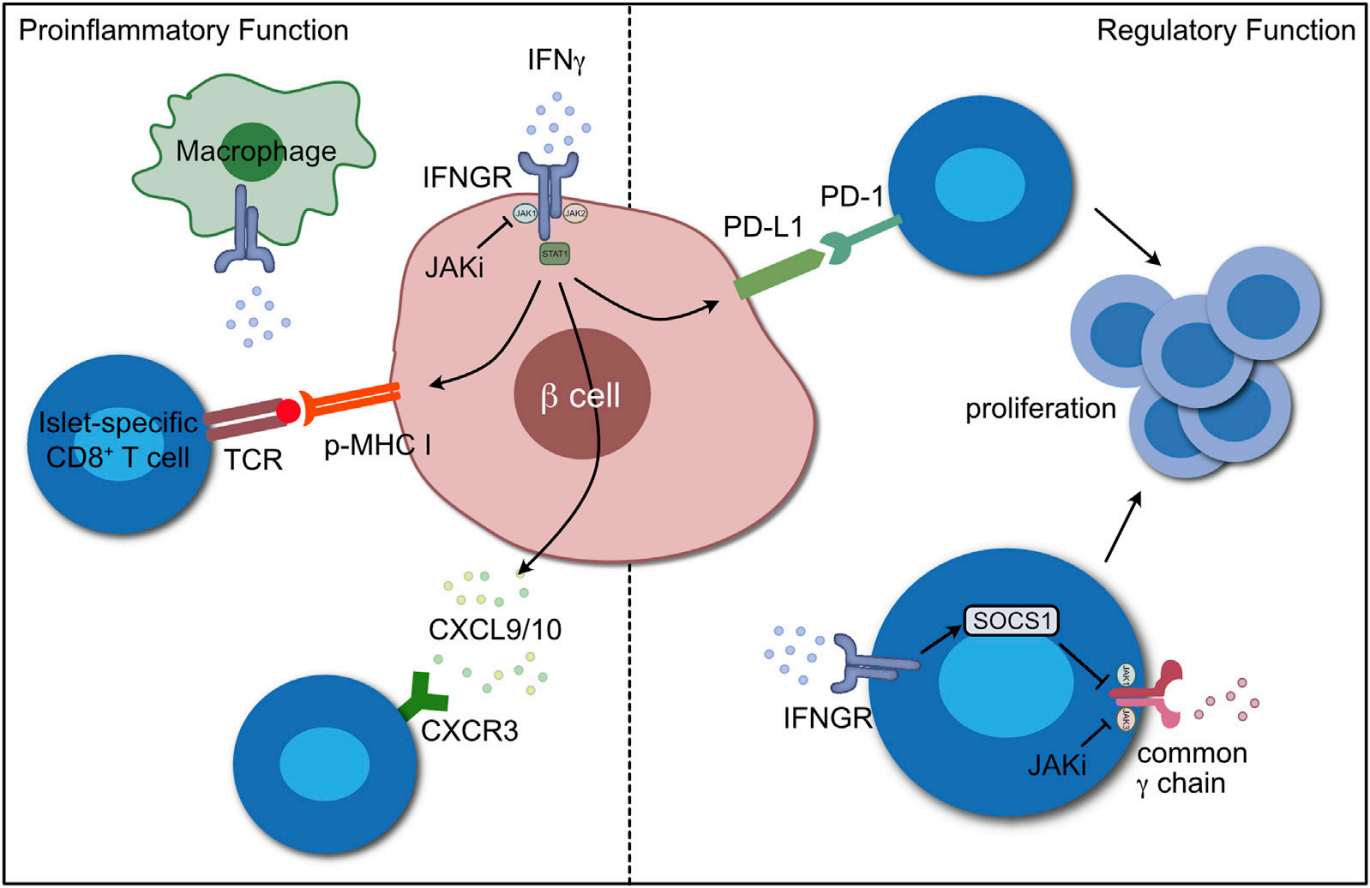
The proinflammatory and regulatory functions of IFN-γ in T1D. The proinflammatory roles (left) include upregulation of MHC class I on the *ß* cell to increase recognition by CD8^+^ T cells, CXCL9 and CXCL10 production to recruit T cells to the islets and enhancing the proinflammatory function of macrophages. The regulatory roles of IFN-γ (right) include upregulation of the checkpoint molecule, PD-L1, in *ß* cells and upregulation of SOCS1 in CD8^+^ T cells to control signaling through common γ chain cytokines. Both of these limit T cell proliferation. JAK inhibitors can block both proinflammatory *ß* cell IFN-γ signaling and γc cytokine responses in T cells to limit *ß* cell destruction. The figure was made using Affinity Designer version 1.10.6.

Interferon-γ (IFN-γ) is produced by T cells, NK cells and other cells of the immune system. It is the classical cytokine of a T helper type 1 (Th1), pro-inflammatory immune response and is often used as a measure of T cell function. IFN-γ is present in the inflammatory islet lesion and has been proposed as a therapeutic target in T1D. However, studies in NOD mice have demonstrated that loss of IFN-γ signaling alone is insufficient to prevent spontaneous autoimmune-driven *ß* cell destruction (Thomas et al., 2009a). We propose that this is because the regulatory roles played by IFN-γ in autoimmune diabetes are also lost. The purpose of this manuscript is to review the pathogenic and protective roles played by IFN-γ in CD8^+^ T cell mediated *ß* cell destruction in autoimmune diabetes and how these roles may be inhibited by blocking the signaling downstream of multiple cytokine receptors with JAK inhibitors (Figure 1).

## 2 Interferons and their signaling pathway

There are three types of interferons. The type I IFNs bind to the IFN-α receptor (IFNAR). Type II IFN, or IFN-γ, binds to the IFN-γ receptor (IFNGR), which comprises IFNGR1 and IFNGR2. Type III IFN, or IFN-λ, binds to IFN-λ receptor 1 (IFNLR1) and IL-10 receptor 2 (IL-10R2). Interferons signal through the JAK-STAT pathway (Philips et al., 2022).

The JAK-STAT signaling pathway relies on two essential components, JAKs and STATs. There are four JAKs, namely, JAK1, JAK2, JAK3, and TYK2 and seven STATs, STAT1, STAT2, STAT3, STAT4, STAT5A, STAT5B and STAT6. Cytokines or hormones bind to their receptors to initiate activation of the JAK tyrosine kinases. Activated JAKs then phosphorylate the receptor tails, which results in the recruitment and activation of STAT proteins. These activated STATs dimerize and enter the nucleus to regulate target gene transcription. Activation of the JAK-STAT pathway is implicated in the development of many diseases, including autoimmune diseases, infection and some cancers (Philips et al., 2022). Multiple cytokines and hormones signal through the JAK-STAT pathway, including IFNs, interleukins, blood cell growth factors and colony-stimulating factors. Cytokines that function through JAK-STAT signaling mediate different downstream events, including cell proliferation, survival, homeostasis, hematopoiesis, inflammation, infection control and tumor surveillance (Kiu and Nicholson, 2012; Leonard et al., 2019).

To prevent excess cytokine signaling, regulators of the pathway play an essential role. These include protein inhibitors of activated STATs (PIAS), protein tyrosine phosphatases (PTPs) and suppressors of cytokine signaling (SOCS). PIAS can bind to activated STAT dimers and suppress STAT-regulated transcription. PTPs can dephosphorylate tyrosine residues on JAKs and STATs to stop the JAK-STAT signal transduction (Gurzov et al., 2015). SOCS can inhibit the JAK-STAT pathway by working as pseudo-JAK substrates and directly binding to JAKs, blocking STAT activation and causing ubiquitin-mediated proteasomal degradation (Liau et al., 2018).

## 3 The pro-inflammatory role of IFN-γ

Our understanding of the immune response within the islets highlights the importance of CD8^+^ T cell recruitment into islets and exposure to *ß* cell antigens in the inflammatory islet microenvironment as a major driver of CD8^+^ T cell proliferation and cytotoxic function (Graham et al., 2011; Sutherland et al., 2017). The capacity of IFN-γ to regulate both CD8^+^ T cell homing and *ß* cell antigen presentation allows it to contribute significantly to the autoreactive CD8^+^ T cell pool. Overexpression of IFN-γ in *ß* cells using the rat insulin promoter (RIP) resulted in diabetes development in BALB/c mice that are not genetically susceptible, demonstrating the capacity of IFN-γ to drive an autoimmune pathology (Sarvetnick et al., 1988).

### 3.1 Cell death

*In vitro*, IFN-γ in combination with IL-1β and/or TNF-α induce *ß* cell apoptosis (Eizirik et al., 2020). This is by a mechanism that involves production of iNOS and activation of intrinsic apoptotic signaling (Thomas et al., 2009b; Eizirik et al., 2020). However, research in NOD mice indicates that blocking the cell death pathways induced by cytokines does not prevent autoimmune diabetes (Thomas et al., 2012). This has been shown in NOD mice deficient in IL-1 receptors or treated with IL-1 receptor antagonists (Thomas et al., 2004; Gill et al., 2016), TNF receptors (Kagi et al., 1999; Chee et al., 2011), the pro-apoptotic molecule BIM (Krishnamurthy et al., 2015) and mice lacking IFN-γ signaling pathways (Thomas et al., 1998; Chong et al., 2004), and in clinical trials blocking IL-1β in T1D (Moran et al., 2013; Cabrera et al., 2015). Therefore, while cytokine induced *ß* cell death is a good marker of cytokine action on *ß* cells, it does not appear to be a good target for T1D prevention.

### 3.2 Homing

IFN-γ is important for homing of autoreactive CD8^+^ T cells into islets by upregulating expression of chemokine receptors on CD8^+^ T cells, including effector-associated CXCR3, and its ligands, such as CXCL10, on *ß* cells (Christen and Kimmel, 2020). Islets display a strong IFN-regulated gene signature at the time of immune cell infiltration, including upregulation of *Cxcl10*, *Cxcl9* and *Cxcl11* (Calderon et al., 2011; Derr et al., 2023). Blocking CXCL10 reduces diabetes (Christen et al., 2003; Christen and Kimmel, 2020) and splenocytes from diabetic NOD mice were less efficient at transferring diabetes into IFN-γ or IFNGR-deficient NOD mice due to impaired homing of T cells into islets (Savinov et al., 2001). Adhesion molecules expressed on endothelial cells in the islet also facilitate extravasation of integrin-expressing autoreactive cells into islets. ICAM-1, MAdCAM-1 and VCAM-1 are expressed on the islet endothelial cells and are upregulated by IFN-γ (Hanninen et al., 1993; Christen et al., 2003; Calderon et al., 2011; Jhala et al., 2022). IFN-γ also upregulates MHC class II on islet endothelial cells that is a hallmark of early islet infiltration in NOD mice, but not required for migration of CD4^+^ T cells into islets (Savinov et al., 2001; Scott et al., 2018).

In addition to T cell recruitment, IFN-γ produced by activated T cells is important for driving recruitment and activation of inflammatory, monocyte-derived macrophages into NOD islets, which maintain the proinflammatory islet microenvironment (Calderon et al., 2008; Hu et al., 2020).

### 3.3 Antigen presentation

IFN-γ facilitates recognition of *ß* cells by increasing the expression of MHC class I on professional antigen presenting cells (APC) and on *ß* cells. Upregulation of MHC class I on *ß* cells is seen in both human T1D and mouse models of the disease (Kay et al., 1991; Richardson et al., 2016). Increased HLA-I expression on *ß* cells in human T1D correlated with upregulation of STAT1, the transcription factor downstream of IFN-γ signaling (Richardson et al., 2016). We showed that inhibiting *ß* cell responsiveness to IFN-γ with a dominant negative IFNGR or overexpression of suppressor of cytokine signaling 1 (SOCS1) in *ß* cells under control of the rat insulin promotor (NOD.RIPSOCS1 mice), blocked upregulation of MHC class I on *ß* cells of NOD mice (Thomas et al., 1998; Chong et al., 2004; Dudek et al., 2006). While type I IFNs can upregulate MHC class I on *ß* cells *in vitro* (Coomans de Brachene et al., 2018; Colli et al., 2020), the extent is less than IFN-γ and at least in NOD mice there is little evidence of a major contribution of type I IFNs to upregulation of MHC class I *in vivo* (Quah et al., 2014).

MHC class I is important for presentation of *ß* cell peptide antigens to CD8^+^ T cells through their autoreactive TCR. Data from our group and others using models with decreased MHC class I expression on the *ß* cell have shown that antigen presentation by *ß* cells accounts for a significant amount of T cell proliferation (Hamilton-Williams et al., 2003; Yamanouchi et al., 2003; Graham et al., 2012; Sutherland et al., 2017). In these studies, *ß* cell specific antigen presentation was reduced by SOCS1 overexpression. Overexpression of SOCS1 blocks IFN-γ signaling in *ß* cells by inhibiting signaling through the JAK-STAT pathway (Chong et al., 2001). T cell proliferation was measured *in vitro* or *in vivo* with transferred or endogenous T cells (Chong et al., 2004; Graham et al., 2012). These experiments showed that antigen specific CD8^+^ T cells in islets of SOCS1 transgenic mice proliferate at a lower rate (BrdU incorporation) and to a lesser extent (CFSE dilution) when compared with wild-type NOD islet T cells (Graham et al., 2012). However, even with low *ß* cell MHC class I, the rate of proliferation in islets remains higher than in the draining pancreatic lymph node. This suggests there is significant antigen presentation from other APCs, which have specialized molecular machinery for antigen presentation and maintain high levels of MHC class I expression even in the absence of IFN-γ signaling. In addition, the inflammatory environment of the islet is awash with growth factors and cytokines that promote T cell proliferation (Carrero et al., 2013).

NOD.RIPSOCS1 mice were completely protected from CD8^+^ T cell mediated diabetes in NOD8.3 TCR transgenic mice and had reduced spontaneous diabetes in NOD mice (Flodstrom-Tullberg et al., 2003; Chong et al., 2004). Similarly, knockout of STAT1, the transcription factor downstream of IFN-γ signaling, was completely protective in NOD mice, and this was attributed to reduced *ß* cell antigen presentation and defects in CD8^+^ T cell development (Kim et al., 2007; Quigley et al., 2008).

Confirming the need for *ß* cell antigen presentation, *ß*-bald mice with *ß* cell specific deletion of beta 2-microglobulin, the light chain for MHC class I, have islet immune cell infiltration (insulitis) equivalent to that in wild-type NOD mice even without the contribution of *ß* cell driven T cell expansion (Hamilton-Williams et al., 2003). Despite this, *ß*-bald mice were significantly protected from spontaneous diabetes indicating reduced *ß* cell destruction as a result of lack of *ß* cell recognition (Hamilton-Williams et al., 2003; Trivedi et al., 2016). It is possible that insulitis differences do exist given the reduced intra-islet proliferation observed in these mice, but that they are masked in the standard five step H&E histology grading system for insulitis. This is a blunt way of observing quantitative changes in insulitis and more recent approaches such as counting CD45^+^ cells or tetramer binding cells in the whole pancreas by flow cytometry may more sensitively demonstrate changes.

The effects of IFN-γ on homing and antigen presentation suggest that IFN-γ plays a disease significant role in autoimmune diabetes. However, multiple groups have demonstrated that deletion of IFN-γ or its receptor does not impact spontaneous diabetes development in the NOD mouse (Hultgren et al., 1996; Kanagawa et al., 2000; Serreze et al., 2000; Carrero et al., 2018; Jhala et al., 2022). This suggests that there is either redundancy between interferons or regulatory roles played by IFN-γ. In both NOD.RIPSOCS1 and STAT1 deficient NOD mice which are significantly protected from diabetes, signaling through all interferon receptors was inhibited, supporting the potential for redundancy in pathogenic function between interferons in autoimmune diabetes (Flodstrom-Tullberg et al., 2003; Chong et al., 2004; Kim et al., 2007). To look at redundancy between interferons, NOD mice deficient in multiple interferon receptors have been made. Deletion of IFNAR and IFNGR reduced and delayed diabetes progression (Carrero et al., 2018; Jhala et al., 2022) which was also seen when all three IFN receptors (IFNAR, IFNGR and IFNLR) were deleted (Jhala et al., 2022). However, diabetes still occurred in the double or triple interferon receptor knockout mice, indicating that there is only minor overlap in function between type I, II and III interferons in autoimmune diabetes.

In summary, in the absence of whole body IFN-γ signaling, there was reduced homing of T cells into islets, reduced *ß* cell antigen presentation and reduced ability of CD8^+^ T cells to recognize and kill *ß* cells and yet, diabetes developed normally in these mice. This was not due to redundancy between interferons. The data indicate that IFN-γ mediated regulatory processes are blocked in the knockout mice, and these will be explored in the next section. The data also indicate that on its own, blockade of IFN-γ signaling globally is not a useful therapeutic target for T1D prevention.

## 4 IFN-γ mediated regulatory processes

### 4.1 T cell proliferation

One way that IFNGR deficiency might promote diabetes is by IFN-γ acting as a brake on T cell proliferation. Several studies in tumor models and in NOD mice have indicated that this is the case. In an engrafted melanoma mouse model, IFN-γ signaling in tumor CD8^+^ T cells restricted anti-tumor responses due to restraint of stem-like exhausted CD8^+^ T cells (Mazet et al., 2023). This population of progenitor exhausted CD8^+^ T cells has also been described in NOD mouse islets and is thought to be the cell population that drives *ß* cell destruction by giving rise to cytotoxic exhausted intermediates which express cytotoxic effector molecules (Zakharov et al., 2020; Ciecko et al., 2021; Grebinoski et al., 2022).

Diabetogenic AI4 CD8^+^ T cells transferred diabetes in IFN-γ-deficient but not wild-type NOD recipients, indicating that IFN-γ functionally suppressed these T cells. This was by a mechanism that involved upregulation of STAT1 expression in the transferred T cells (Driver et al., 2017). Our lab showed that IFNGR deficient NOD mice had a 10-fold increase in the number of islet antigen specific CD8^+^ T cells, also indicating that IFN-γ signaling restricts the expansion of these cells (Jhala et al., 2022). We demonstrated that the T cells expand because of increased sensitivity to common γ chain (γc) cytokines, particularly IL-2. This was at least in part due to a reduction in SOCS1 expression, which is regulated by IFN-γ in a feedback mechanism and regulates JAK-STAT dependent cytokine signaling. This role for SOCS1 in controlling T cell proliferation is consistent with previous reports in SOCS1 deficient mice (Alexander et al., 1999; Metcalf et al., 2002).

This promotion of autoimmune diabetes development with an increase in γc cytokine signaling has been shown in other settings. Humans with heterozygous gain-of-function mutations in STAT1 or STAT3, associated with γc signaling, are prone to developing T1D (Flanagan et al., 2014; Toubiana et al., 2016; Velayos et al., 2017). A recent study suggests that the gain-of-function mutation in STAT3 drives expansion of effector CD8^+^ T cells when expressed in NOD mice, resulting in accelerated diabetes (Warshauer et al., 2021). Protein tyrosine phosphatase non-receptor type 2 (PTPN2) is expressed in T cells and attenuates cytokine signaling. PTPN2 deficiency in T cells increases TCR sensitivity and enhances responses to γc cytokines (Fukushima et al., 2010; Wiede et al., 2011; Zikherman and Weiss, 2011), and these cells have increased expansion and survival capacity (Flosbach et al., 2020) that leads to accelerated diabetes in NOD mice (Wiede et al., 2019). While these studies do not involve loss of IFN-γ signaling, they demonstrate that increasing proliferation and thus greater numbers of antigen specific T cells can promote progression to diabetes. Thus, the expanded islet-specific CD8^+^ T cells seen with IFN-γ signaling deficiency likely compensate for the reduced proinflammatory effects of IFN-γ within the islets of IFN-γ or IFNGR deficient mice.

### 4.2 Immune checkpoints

IFN-γ is the primary driver of *ß* cell PD-L1 expression and upregulates PD-L1 on APCs and other islet cells (Colli et al., 2018; Osum et al., 2018). PD-L1 ligates with PD-1 on activated T cells as an important immune checkpoint to limit destruction of host tissues during chronic antigen exposure. The PD-1/PD-L1 axis is known to limit CD8^+^ T cell-mediated *ß* cell destruction (Ansari et al., 2003; Pauken et al., 2013). NOD mice treated with anti-PD-L1 or anti-PD-1 antibodies develop rapid diabetes associated with increased CD8^+^ T cell proliferation (Ansari et al., 2003; Pauken et al., 2013).

Thus, IFN-γ acts to limit the CD8^+^ T cell pool in autoimmune diabetes by regulating responses to pro-T-cell-survival cytokines and limiting antigen-dependent expansion through *ß* cell immune checkpoint induction. Together these results indicate that there are differential effects of IFN-γ signaling on autoimmune diabetic processes in *ß* cells and T cells that result in the surprising lack of protection in IFN-γ and IFNGR deficient NOD mice.

## 5 JAK inhibitors

JAK inhibitors, which block multiple cytokines functioning through the JAK-STAT pathway, were developed for inflammatory and autoimmune diseases (Tanaka et al., 2022). JAK inhibitors work by impeding ATP binding required for tyrosine kinase-mediated protein phosphorylation (Liau et al., 2018; Tanaka et al., 2022). Many JAK inhibitors have been developed. They are classified into first-generation JAK inhibitors, or pan-JAK inhibitors, which work against most or all JAK members and second-generation JAK inhibitors, which selectively inhibit a particular JAK or subset of JAKs. There are now at least five JAK inhibitors approved for treatment of various inflammatory diseases including rheumatological diseases, ulcerative colitis, atopic dermatitis and alopecia areata. A new generation of allosteric JAK inhibitors that show greater selectivity for individual JAK molecules are now in development (Kavanagh et al., 2022).

JAK inhibitors have a demonstrated safety and are well-tolerated (Winthrop, 2017; Winthrop and Cohen, 2022). JAK1/JAK2 inhibitors transiently reduce white blood cell numbers and hemoglobulin levels due to inhibition of JAK2 dependent blood cell development cytokines. Selective JAK inhibitors impact restricted cytokines, thus reducing the side effects caused by inhibiting other JAKs. Other side effects include an increase in common infections and shingles, seen in approximately 4 cases per 100 patient years (Smolen et al., 2019). In patients with high risk, for example, in smokers, an increased risk of cardiovascular disease and certain cancers has been shown for some JAK inhibitors (Winthrop and Cohen, 2022).

### 5.1 JAK inhibitors in T1D

The use of JAK inhibitors has been studied in the context of T1D. As expected, JAK inhibitors block *ß* cell death induced by proinflammatory cytokines and prevent IFN-γ mediated upregulation of MHC class I on *ß* cells (Trivedi et al., 2017; Colli et al., 2020). Because of the wide range of cytokines that signal using the JAK-STAT pathway and the diversity of cytokine receptor expression, JAK inhibitors have the potential to affect many different cell types in spontaneous diabetes.

In NOD mice, JAK1 selective inhibition blocked γc cytokine signaling in T cells and reduced T cell proliferation and differentiation (Ge et al., 2020). Diminished T cell proliferation caused by JAK inhibition was observed more in CD8^+^ T cells than in CD4^+^ T cells, suggesting a selective dependence of CD8^+^ T cells on JAK-STAT cytokines (Goldrath et al., 2002; Tan et al., 2002). NOD mice treated with JAK inhibitors had reduced MHC class I expression on *ß* cells thus limiting the ability of CD8^+^ T cells to interact with *ß* cells (Trivedi et al., 2017; Ge et al., 2020). The result of the effects of JAK inhibitors on both immune cells and *ß* cells was to prevent and reverse spontaneous diabetes in NOD mice and diabetes induced by the checkpoint inhibitor anti-PD-L1 (Trivedi et al., 2017; Ge et al., 2020; Ge et al., 2022).

The mechanism of action of JAK inhibitors is akin to that of SOCS1, i.e., inhibiting the ability of the JAK to interact with and phosphorylate STATs (Liau et al., 2018). Thus, it is not surprising that overexpression of SOCS1 in beta cells has a similar phenotype to treatment with JAK inhibitor. The difference is that systemic JAK inhibitor treatment also inhibits the response of CD8^+^ T cells to γc cytokines and likely inhibits responses of other immune cells, such as inflammatory macrophages, APCs and NK cells, to cytokines, affecting their ability to contribute to diabetes pathogenesis (Figure 1). The JAK1/JAK2 inhibitor baricitinib is currently being tested in newly diagnosed T1D in the BANDIT clinical trial (NCT04774224), with results expected mid-2023 (Waibel et al., 2022).

## 6 Perspectives

IFN-γ has a role in the pathogenesis of T1D, including facilitating homing of T cells into islets and increasing recognition of *ß* cells by CD8^+^ T cells. Despite this, its inhibition does not prevent autoimmune diabetes in NOD mice. Inhibition of IFN-γ signaling has not been tested in human T1D and our review of the current literature suggests that it would not be a good target. Recent studies showing the role of IFN-γ in controlling antigen specific CD8^+^ T cell proliferation, and the expression of PD-L1, the ligand for the checkpoint molecule PD-1, have shed light on the lack of therapeutic benefit of IFN-γ blockade in mouse models. In contrast, it is important to block the inflammatory effects of IFN-γ while also controlling T cell proliferation. This can be done by blocking multiple cytokines, including interferons and γc cytokines, with JAK inhibitors. We have shown that JAK inhibitors can prevent and reverse autoimmune diabetes and one JAK inhibitor, baricitinib, is currently being tested in the clinic. The ability of JAK inhibitors to affect both *ß* cells and cells of the immune system makes them different to other immune therapies that have been tested in T1D that generally only inhibit the immune system.
